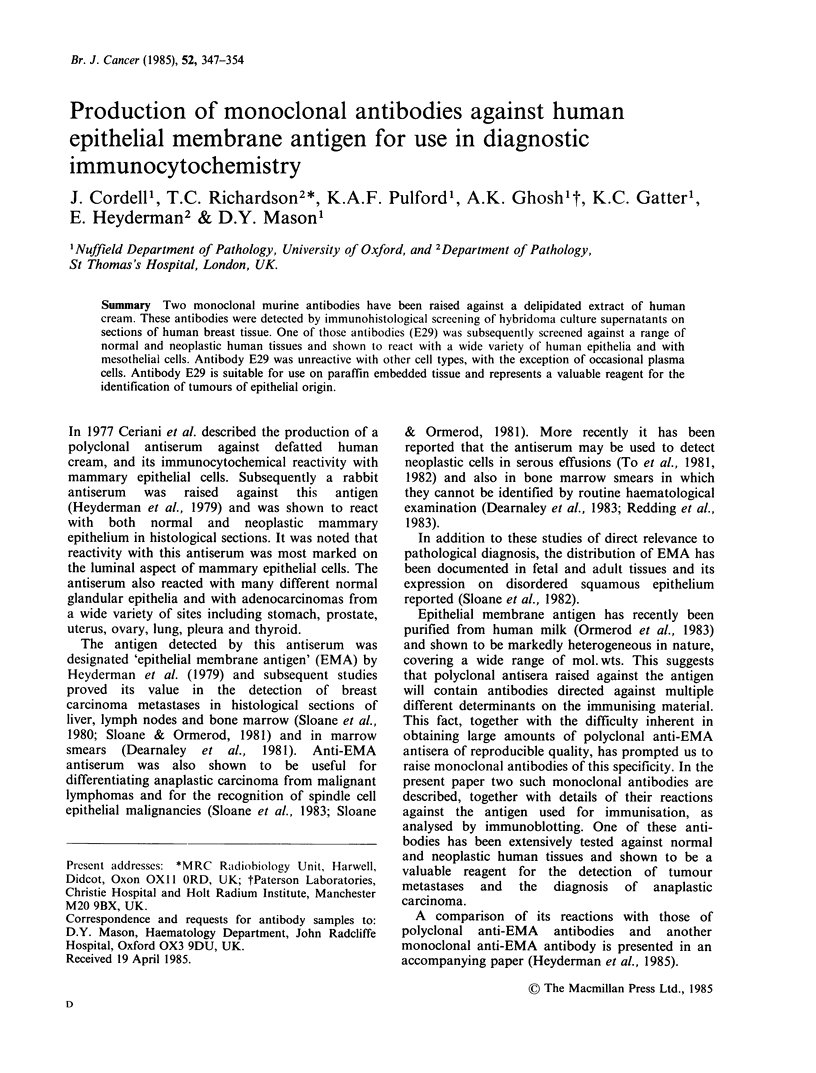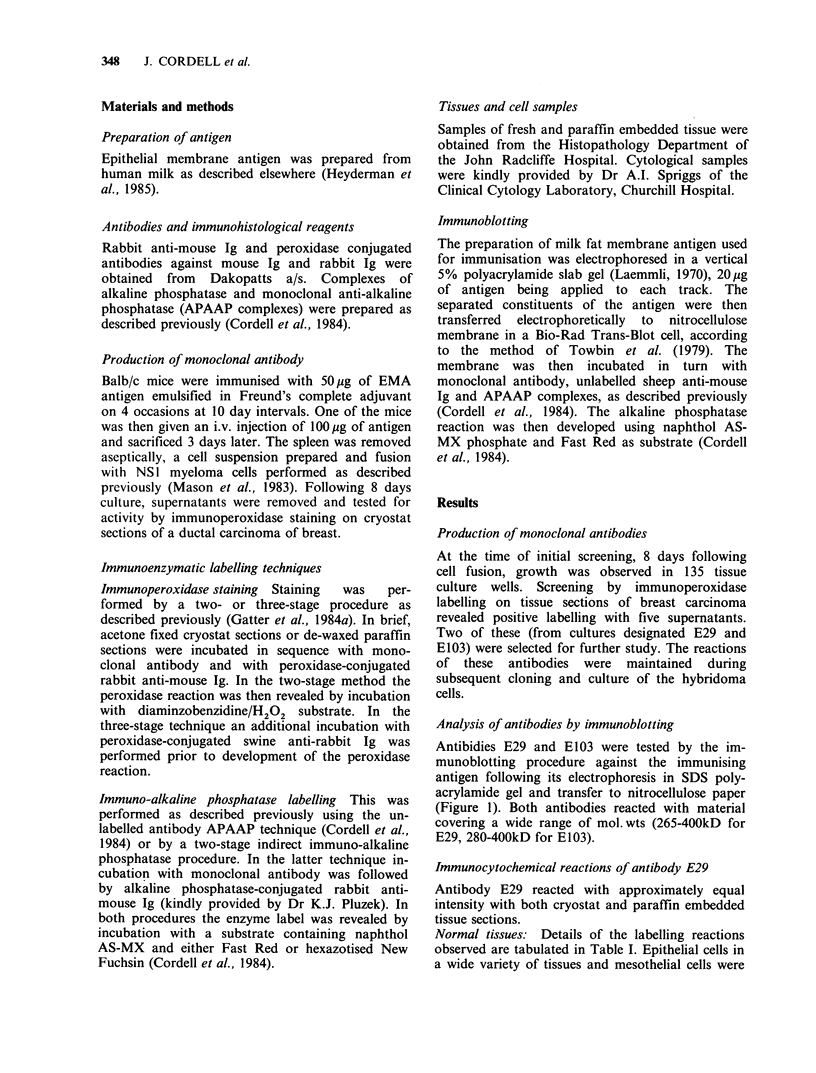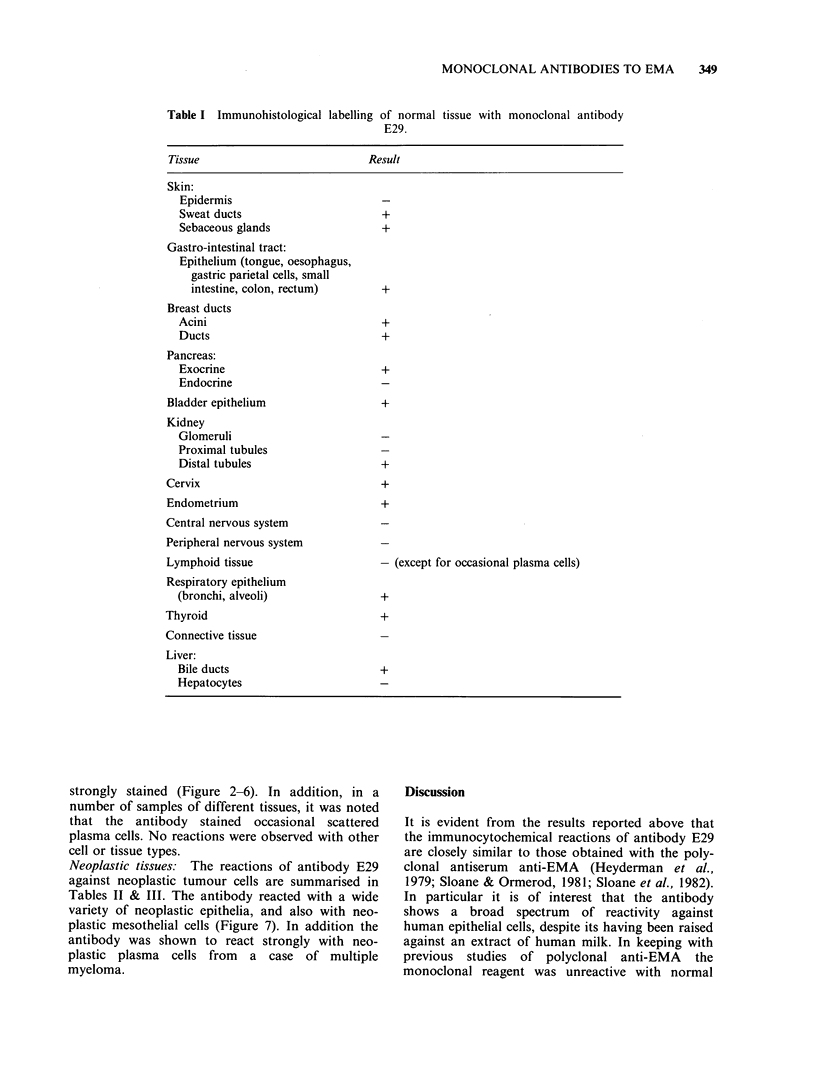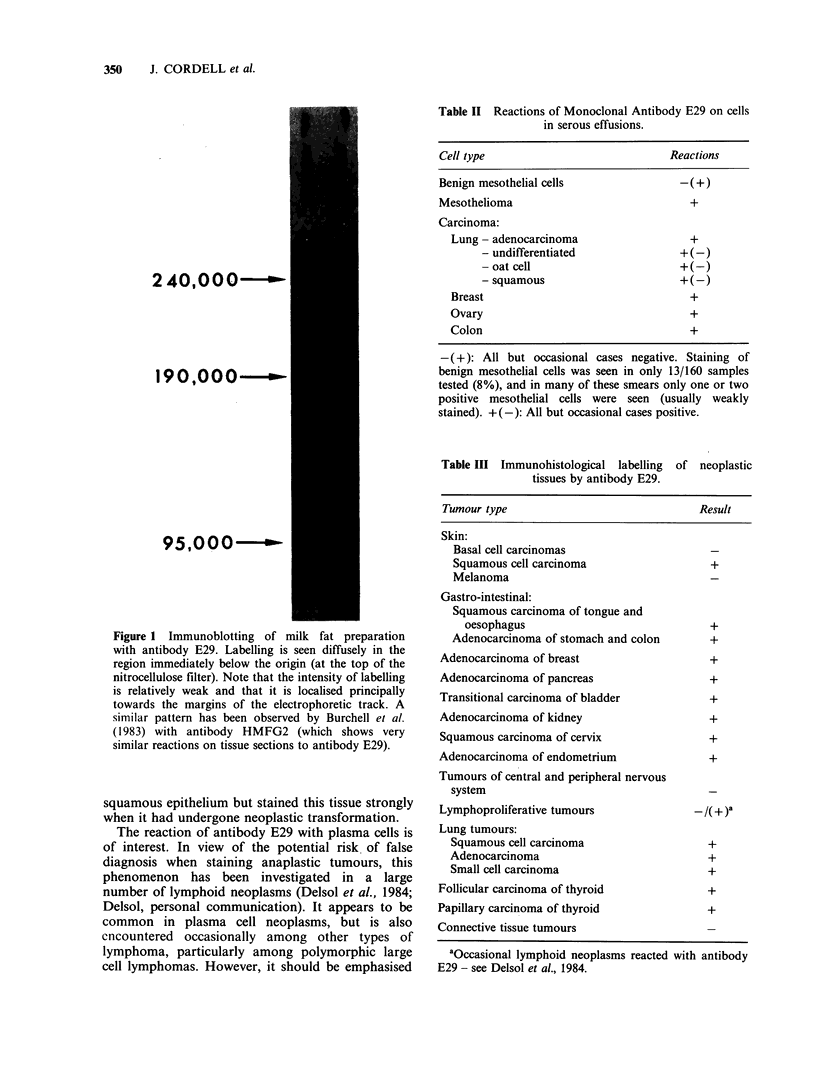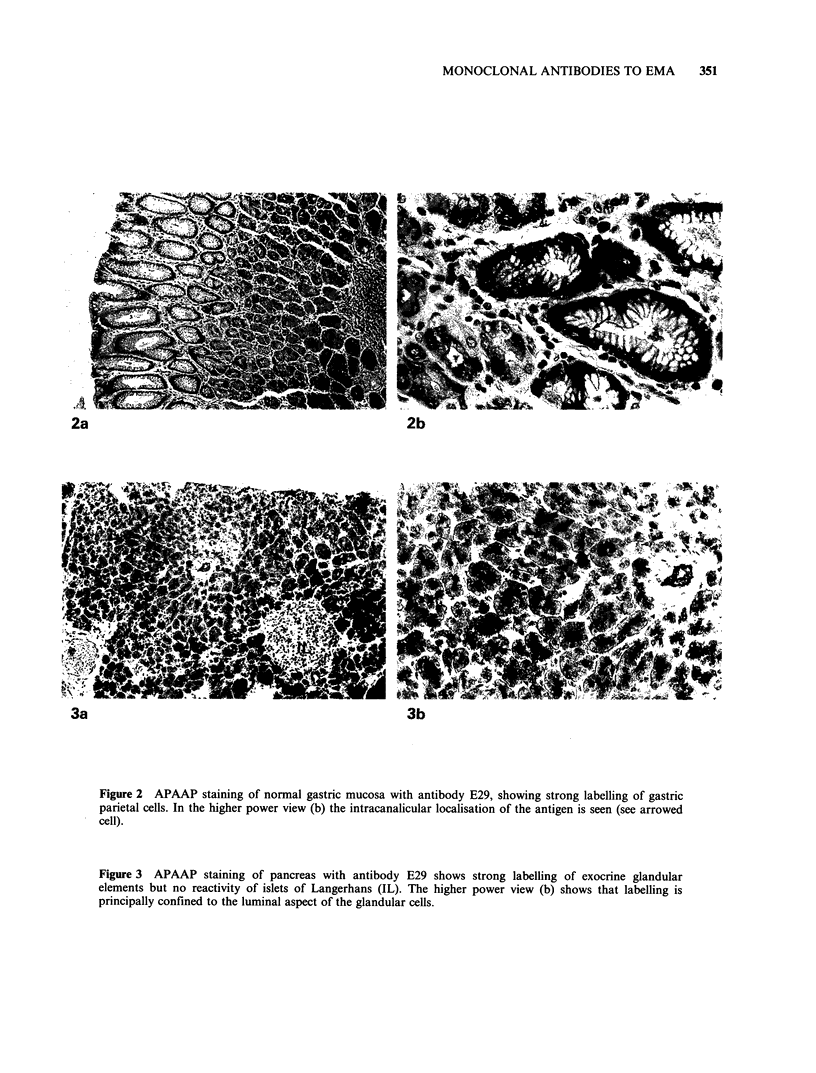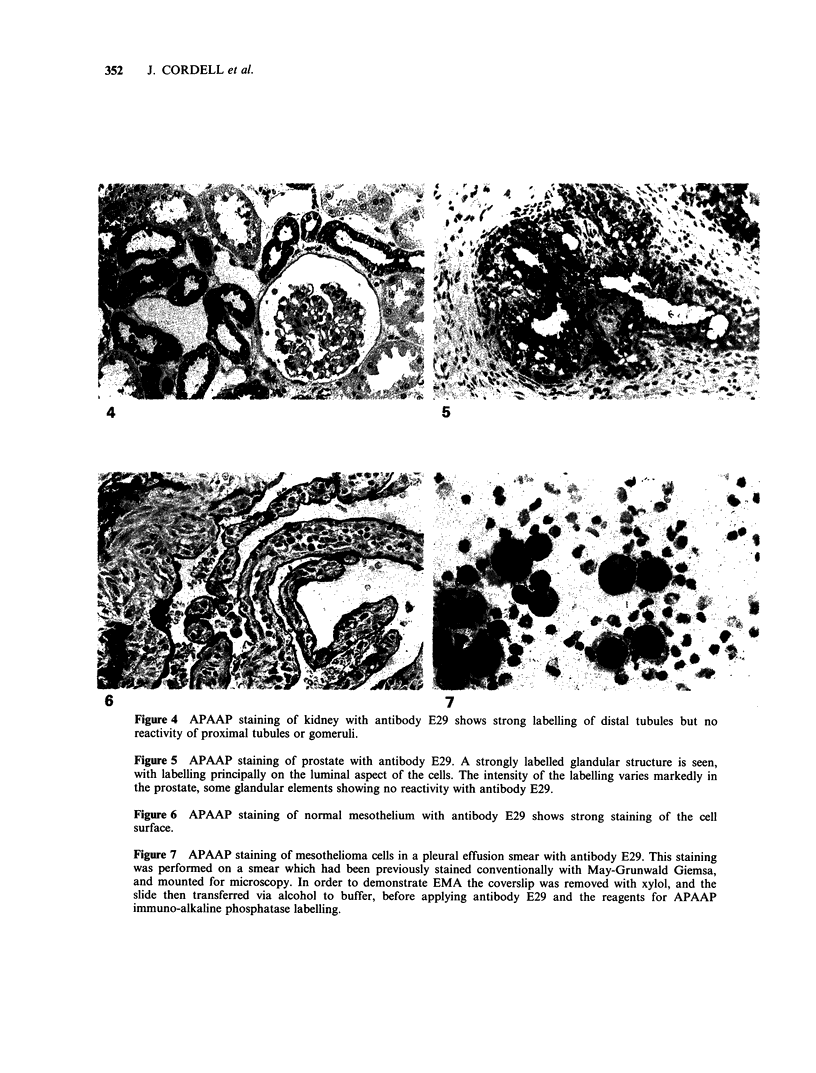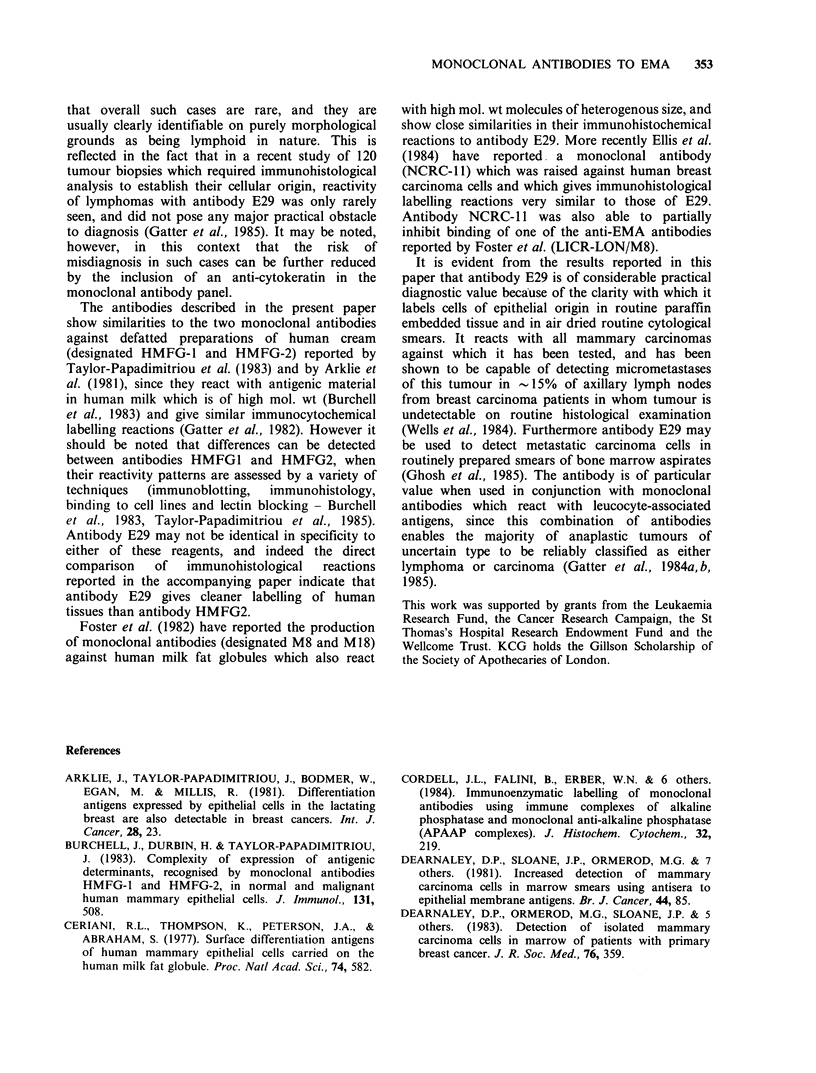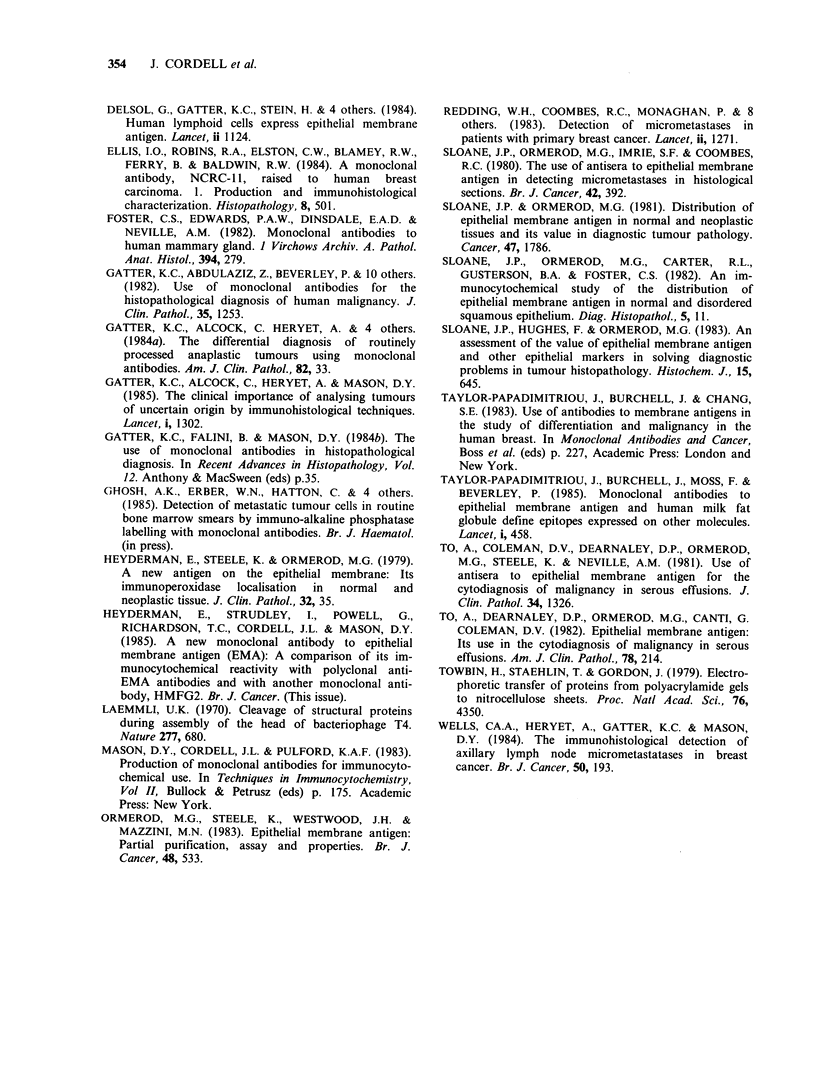# Production of monoclonal antibodies against human epithelial membrane antigen for use in diagnostic immunocytochemistry.

**DOI:** 10.1038/bjc.1985.200

**Published:** 1985-09

**Authors:** J. Cordell, T. C. Richardson, K. A. Pulford, A. K. Ghosh, K. C. Gatter, E. Heyderman, D. Y. Mason

## Abstract

**Images:**


*Br. J. Cancer* (1985), **52**, 347–354

### Production of monoclonal antibodies against human epithelial membrane antigen for use in diagnostic immunocytochemistry

J. Cordell[1], T.C. Richardson[2*], K.A.F. Pulford[1], A.K. Ghosh[1†], K.C. Gatter[1], E. Heyderman[2] & D.Y. Mason[1]

[1]*Nuffield Department of Pathology, University of Oxford, and* [2]*Department of Pathology, St Thomas's Hospital, London, UK.*

**Summary** Two monoclonal murine antibodies have been raised against a delipidated extract of human cream. These antibodies were detected by immunohistological screening of hybridoma culture supernatants on sections of human breast tissue. One of those antibodies (E29) was subsequently screened against a range of normal and neoplastic human tissues and shown to react with a wide variety of human epithelia and with mesothelial cells. Antibody E29 was unreactive with other cell types, with the exception of occasional plasma cells. Antibody E29 is suitable for use on paraffin embedded tissue and represents a valuable reagent for the identification of tumours of epithelial origin.

In 1977 Ceriani *et al.* described the production of a polyclonal antiserum against defatted human cream, and its immunocytochemical reactivity with mammary epithelial cells. Subsequently a rabbit antiserum was raised against this antigen (Heyderman *et al.*, 1979) and was shown to react with both normal and neoplastic mammary epithelium in histological sections. It was noted that reactivity with this antiserum was most marked on the luminal aspect of mammary epithelial cells. The antiserum also reacted with many different normal glandular epithelia and with adenocarcinomas from a wide variety of sites including stomach, prostate, uterus, ovary, lung, pleura and thyroid.

The antigen detected by this antiserum was designated 'epithelial membrane antigen' (EMA) by Heyderman *et al.* (1979) and subsequent studies proved its value in the detection of breast carcinoma metastases in histological sections of liver, lymph nodes and bone marrow (Sloane *et al.*, 1980; Sloane & Ormerod, 1981) and in marrow smears (Dearnaley *et al.*, 1981). Anti-EMA antiserum was also shown to be useful for differentiating anaplastic carcinoma from malignant lymphomas and for the recognition of spindle cell epithelial malignancies (Sloane *et al.*, 1983; Sloane & Ormerod, 1981). More recently it has been reported that the antiserum may be used to detect neoplastic cells in serous effusions (To *et al.*, 1981, 1982) and also in bone marrow smears in which they cannot be identified by routine haematological examination (Dearnaley *et al.*, 1983; Redding *et al.*, 1983).

In addition to these studies of direct relevance to pathological diagnosis, the distribution of EMA has been documented in fetal and adult tissues and its expression on disordered squamous epithelium reported (Sloane *et al.*, 1982).

Epithelial membrane antigen has recently been purified from human milk (Ormerod *et al.*, 1983) and shown to be markedly heterogeneous in nature, covering a wide range of mol. wts. This suggests that polyclonal antisera raised against the antigen will contain antibodies directed against multiple different determinants on the immunising material. This fact, together with the difficulty inherent in obtaining large amounts of polyclonal anti-EMA antisera of reproducible quality, has prompted us to raise monoclonal antibodies of this specificity. In the present paper two such monoclonal antibodies are described, together with details of their reactions against the antigen used for immunisation, as analysed by immunoblotting. One of these antibodies has been extensively tested against normal and neoplastic human tissues and shown to be a valuable reagent for the detection of tumour metastases and the diagnosis of anaplastic carcinoma.

A comparison of its reactions with those of polyclonal anti-EMA antibodies and another monoclonal anti-EMA antibody is presented in an accompanying paper (Heyderman *et al.*, 1985).

---

Present addresses: *MRC Radiobiology Unit, Harwell, Didcot, Oxon OX11 0RD, UK; †Paterson Laboratories, Christie Hospital and Holt Radium Institute, Manchester M20 9BX, UK.
Correspondence and requests for antibody samples to: D.Y. Mason, Haematology Department, John Radcliffe Hospital, Oxford OX3 9DU, UK.
Received 19 April 1985.

© The Macmillan Press Ltd., 1985

### Materials and methods

### *Preparation of antigen*

Epithelial membrane antigen was prepared from human milk as described elsewhere (Heyderman *et al.*, 1985).

### *Antibodies and immunohistological reagents*

Rabbit anti-mouse Ig and peroxidase conjugated antibodies against mouse Ig and rabbit Ig were obtained from Dakopatts a/s. Complexes of alkaline phosphatase and monoclonal anti-alkaline phosphatase (APAAP complexes) were prepared as described previously (Cordell *et al.*, 1984).

### *Production of monoclonal antibody*

Balb/c mice were immunised with 50 μg of EMA antigen emulsified in Freund's complete adjuvant on 4 occasions at 10 day intervals. One of the mice was then given an i.v. injection of 100 μg of antigen and sacrificed 3 days later. The spleen was removed aseptically, a cell suspension prepared and fusion with NS1 myeloma cells performed as described previously (Mason *et al.*, 1983). Following 8 days culture, supernatants were removed and tested for activity by immunoperoxidase staining on cryostat sections of a ductal carcinoma of breast.

### *Immunoenzymatic labelling techniques*

*Immunoperoxidase staining* Staining was performed by a two- or three-stage procedure as described previously (Gatter *et al.*, 1984*a*). In brief, acetone fixed cryostat sections or de-waxed paraffin sections were incubated in sequence with monoclonal antibody and with peroxidase-conjugated rabbit anti-mouse Ig. In the two-stage method the peroxidase reaction was then revealed by incubation with diaminzobenzidine/$H_2O_2$ substrate. In the three-stage technique an additional incubation with peroxidase-conjugated swine anti-rabbit Ig was performed prior to development of the peroxidase reaction.

*Immuno-alkaline phosphatase labelling* This was performed as described previously using the unlabelled antibody APAAP technique (Cordell *et al.*, 1984) or by a two-stage indirect immuno-alkaline phosphatase procedure. In the latter technique incubation with monoclonal antibody was followed by alkaline phosphatase-conjugated rabbit anti-mouse Ig (kindly provided by Dr K.J. Pluzek). In both procedures the enzyme label was revealed by incubation with a substrate containing naphthol AS-MX and either Fast Red or hexazotised New Fuchsin (Cordell *et al.*, 1984).

### *Tissues and cell samples*

Samples of fresh and paraffin embedded tissue were obtained from the Histopathology Department of the John Radcliffe Hospital. Cytological samples were kindly provided by Dr A.I. Spriggs of the Clinical Cytology Laboratory, Churchill Hospital.

### *Immunoblotting*

The preparation of milk fat membrane antigen used for immunisation was electrophoresed in a vertical 5% polyacrylamide slab gel (Laemmli, 1970), 20 μg of antigen being applied to each track. The separated constituents of the antigen were then transferred electrophoretically to nitrocellulose membrane in a Bio-Rad Trans-Blot cell, according to the method of Towbin *et al.* (1979). The membrane was then incubated in turn with monoclonal antibody, unlabelled sheep anti-mouse Ig and APAAP complexes, as described previously (Cordell *et al.*, 1984). The alkaline phosphatase reaction was then developed using naphthol AS-MX phosphate and Fast Red as substrate (Cordell *et al.*, 1984).

### Results

### *Production of monoclonal antibodies*

At the time of initial screening, 8 days following cell fusion, growth was observed in 135 tissue culture wells. Screening by immunoperoxidase labelling on tissue sections of breast carcinoma revealed positive labelling with five supernatants. Two of these (from cultures designated E29 and E103) were selected for further study. The reactions of these antibodies were maintained during subsequent cloning and culture of the hybridoma cells.

### *Analysis of antibodies by immunoblotting*

Antibidies E29 and E103 were tested by the immunoblotting procedure against the immunising antigen following its electrophoresis in SDS polyacrylamide gel and transfer to nitrocellulose paper (Figure 1). Both antibodies reacted with material covering a wide range of mol. wts (265-400kD for E29, 280-400kD for E103).

### *Immunocytochemical reactions of antibody E29*

Antibody E29 reacted with approximately equal intensity with both cryostat and paraffin embedded tissue sections.

*Normal tissues:* Details of the labelling reactions observed are tabulated in Table I. Epithelial cells in a wide variety of tissues and mesothelial cells were

**Table I** Immunohistological labelling of normal tissue with monoclonal antibody E29.

| *Tissue* | *Result* |
|---|---|
| Skin: | |
| Epidermis | – |
| Sweat ducts | + |
| Sebaceous glands | + |
| Gastro-intestinal tract: | |
| Epithelium (tongue, oesophagus, gastric parietal cells, small intestine, colon, rectum) | + |
| Breast ducts | |
| Acini | + |
| Ducts | + |
| Pancreas: | |
| Exocrine | + |
| Endocrine | – |
| Bladder epithelium | + |
| Kidney | |
| Glomeruli | – |
| Proximal tubules | – |
| Distal tubules | + |
| Cervix | + |
| Endometrium | + |
| Central nervous system | – |
| Peripheral nervous system | – |
| Lymphoid tissue | – (except for occasional plasma cells) |
| Respiratory epithelium (bronchi, alveoli) | + |
| Thyroid | + |
| Connective tissue | – |
| Liver: | |
| Bile ducts | + |
| Hepatocytes | – |

strongly stained (Figure 2–6). In addition, in a number of samples of different tissues, it was noted that the antibody stained occasional scattered plasma cells. No reactions were observed with other cell or tissue types.

*Neoplastic tissues:* The reactions of antibody E29 against neoplastic tumour cells are summarised in Tables II & III. The antibody reacted with a wide variety of neoplastic epithelia, and also with neoplastic mesothelial cells (Figure 7). In addition the antibody was shown to react strongly with neoplastic plasma cells from a case of multiple myeloma.

### Discussion

It is evident from the results reported above that the immunocytochemical reactions of antibody E29 are closely similar to those obtained with the polyclonal antiserum anti-EMA (Heyderman *et al.*, 1979; Sloane & Ormerod, 1981; Sloane *et al.*, 1982). In particular it is of interest that the antibody shows a broad spectrum of reactivity against human epithelial cells, despite its having been raised against an extract of human milk. In keeping with previous studies of polyclonal anti-EMA the monoclonal reagent was unreactive with normal

**Figure 1** Immunoblotting of milk fat preparation with antibody E29. Labelling is seen diffusely in the region immediately below the origin (at the top of the nitrocellulose filter). Note that the intensity of labelling is relatively weak and that it is localised principally towards the margins of the electrophoretic track. A similar pattern has been observed by Burchell *et al.* (1983) with antibody HMFG2 (which shows very similar reactions on tissue sections to antibody E29).

squamous epithelium but stained this tissue strongly when it had undergone neoplastic transformation.

The reaction of antibody E29 with plasma cells is of interest. In view of the potential risk of false diagnosis when staining anaplastic tumours, this phenomenon has been investigated in a large number of lymphoid neoplasms (Delsol *et al.*, 1984; Delsol, personal communication). It appears to be common in plasma cell neoplasms, but is also encountered occasionally among other types of lymphoma, particularly among polymorphic large cell lymphomas. However, it should be emphasised

**Table II** Reactions of Monoclonal Antibody E29 on cells in serous effusions.

| *Cell type* | *Reactions* |
|---|---|
| Benign mesothelial cells | −(+) |
| Mesothelioma | + |
| Carcinoma: | |
| Lung – adenocarcinoma | + |
| – undifferentiated | +(−) |
| – oat cell | +(−) |
| – squamous | +(−) |
| Breast | + |
| Ovary | + |
| Colon | + |

−(+): All but occasional cases negative. Staining of benign mesothelial cells was seen in only 13/160 samples tested (8%), and in many of these smears only one or two positive mesothelial cells were seen (usually weakly stained). +(−): All but occasional cases positive.

**Table III** Immunohistological labelling of neoplastic tissues by antibody E29.

| *Tumour type* | *Result* |
|---|---|
| Skin: | |
| Basal cell carcinomas | − |
| Squamous cell carcinoma | + |
| Melanoma | − |
| Gastro-intestinal: | |
| Squamous carcinoma of tongue and oesophagus | + |
| Adenocarcinoma of stomach and colon | + |
| Adenocarcinoma of breast | + |
| Adenocarcinoma of pancreas | + |
| Transitional carcinoma of bladder | + |
| Adenocarcinoma of kidney | + |
| Squamous carcinoma of cervix | + |
| Adenocarcinoma of endometrium | + |
| Tumours of central and peripheral nervous system | − |
| Lymphoproliferative tumours | −/(+)[a] |
| Lung tumours: | |
| Squamous cell carcinoma | + |
| Adenocarcinoma | + |
| Small cell carcinoma | + |
| Follicular carcinoma of thyroid | + |
| Papillary carcinoma of thyroid | + |
| Connective tissue tumours | − |

[a]Occasional lymphoid neoplasms reacted with antibody E29 – see Delsol *et al.*, 1984.

**Figure 2** APAAP staining of normal gastric mucosa with antibody E29, showing strong labelling of gastric parietal cells. In the higher power view (b) the intracanalicular localisation of the antigen is seen (see arrowed cell).

**Figure 3** APAAP staining of pancreas with antibody E29 shows strong labelling of exocrine glandular elements but no reactivity of islets of Langerhans (IL). The higher power view (b) shows that labelling is principally confined to the luminal aspect of the glandular cells.

4

5

6

7

**Figure 4** APAAP staining of kidney with antibody E29 shows strong labelling of distal tubules but no reactivity of proximal tubules or gomeruli.

**Figure 5** APAAP staining of prostate with antibody E29. A strongly labelled glandular structure is seen, with labelling principally on the luminal aspect of the cells. The intensity of the labelling varies markedly in the prostate, some glandular elements showing no reactivity with antibody E29.

**Figure 6** APAAP staining of normal mesothelium with antibody E29 shows strong staining of the cell surface.

**Figure 7** APAAP staining of mesothelioma cells in a pleural effusion smear with antibody E29. This staining was performed on a smear which had been previously stained conventionally with May-Grunwald Giemsa, and mounted for microscopy. In order to demonstrate EMA the coverslip was removed with xylol, and the slide then transferred via alcohol to buffer, before applying antibody E29 and the reagents for APAAP immuno-alkaline phosphatase labelling.

that overall such cases are rare, and they are usually clearly identifiable on purely morphological grounds as being lymphoid in nature. This is reflected in the fact that in a recent study of 120 tumour biopsies which required immunohistological analysis to establish their cellular origin, reactivity of lymphomas with antibody E29 was only rarely seen, and did not pose any major practical obstacle to diagnosis (Gatter *et al.*, 1985). It may be noted, however, in this context that the risk of misdiagnosis in such cases can be further reduced by the inclusion of an anti-cytokeratin in the monoclonal antibody panel.

The antibodies described in the present paper show similarities to the two monoclonal antibodies against defatted preparations of human cream (designated HMFG-1 and HMFG-2) reported by Taylor-Papadimitriou *et al.* (1983) and by Arklie *et al.* (1981), since they react with antigenic material in human milk which is of high mol. wt (Burchell *et al.*, 1983) and give similar immunocytochemical labelling reactions (Gatter *et al.*, 1982). However it should be noted that differences can be detected between antibodies HMFG1 and HMFG2, when their reactivity patterns are assessed by a variety of techniques (immunoblotting, immunohistology, binding to cell lines and lectin blocking – Burchell *et al.*, 1983, Taylor-Papadimitriou *et al.*, 1985). Antibody E29 may not be identical in specificity to either of these reagents, and indeed the direct comparison of immunohistological reactions reported in the accompanying paper indicate that antibody E29 gives cleaner labelling of human tissues than antibody HMFG2.

Foster *et al.* (1982) have reported the production of monoclonal antibodies (designated M8 and M18) against human milk fat globules which also react with high mol. wt molecules of heterogenous size, and show close similarities in their immunohistochemical reactions to antibody E29. More recently Ellis *et al.* (1984) have reported a monoclonal antibody (NCRC-11) which was raised against human breast carcinoma cells and which gives immunohistological labelling reactions very similar to those of E29. Antibody NCRC-11 was also able to partially inhibit binding of one of the anti-EMA antibodies reported by Foster *et al.* (LICR-LON/M8).

It is evident from the results reported in this paper that antibody E29 is of considerable practical diagnostic value because of the clarity with which it labels cells of epithelial origin in routine paraffin embedded tissue and in air dried routine cytological smears. It reacts with all mammary carcinomas against which it has been tested, and has been shown to be capable of detecting micrometastases of this tumour in ~15% of axillary lymph nodes from breast carcinoma patients in whom tumour is undetectable on routine histological examination (Wells *et al.*, 1984). Furthermore antibody E29 may be used to detect metastatic carcinoma cells in routinely prepared smears of bone marrow aspirates (Ghosh *et al.*, 1985). The antibody is of particular value when used in conjunction with monoclonal antibodies which react with leucocyte-associated antigens, since this combination of antibodies enables the majority of anaplastic tumours of uncertain type to be reliably classified as either lymphoma or carcinoma (Gatter *et al.*, 1984*a,b*, 1985).

This work was supported by grants from the Leukaemia Research Fund, the Cancer Research Campaign, the St Thomas's Hospital Research Endowment Fund and the Wellcome Trust. KCG holds the Gillson Scholarship of the Society of Apothecaries of London.

### References

ARKLIE, J., TAYLOR-PAPADIMITRIOU, J., BODMER, W., EGAN, M. & MILLIS, R. (1981). Differentiation antigens expressed by epithelial cells in the lactating breast are also detectable in breast cancers. *Int. J. Cancer*, **28**, 23.

BURCHELL, J., DURBIN, H. & TAYLOR-PAPADIMITRIOU, J. (1983). Complexity of expression of antigenic determinants, recognised by monoclonal antibodies HMFG-1 and HMFG-2, in normal and malignant human mammary epithelial cells. *J. Immunol.*, **131**, 508.

CERIANI, R.L., THOMPSON, K., PETERSON, J.A., & ABRAHAM, S. (1977). Surface differentiation antigens of human mammary epithelial cells carried on the human milk fat globule. *Proc. Natl Acad. Sci.*, **74**, 582.

CORDELL, J.L., FALINI, B., ERBER, W.N. & 6 others. (1984). Immunoenzymatic labelling of monoclonal antibodies using immune complexes of alkaline phosphatase and monoclonal anti-alkaline phosphatase (APAAP complexes). *J. Histochem. Cytochem.*, **32**, 219.

DEARNALEY, D.P., SLOANE, J.P., ORMEROD, M.G. & 7 others. (1981). Increased detection of mammary carcinoma cells in marrow smears using antisera to epithelial membrane antigens. *Br. J. Cancer*, **44**, 85.

DEARNALEY, D.P., ORMEROD, M.G., SLOANE, J.P. & 5 others. (1983). Detection of isolated mammary carcinoma cells in marrow of patients with primary breast cancer. *J. R. Soc. Med.*, **76**, 359.

DELSOL, G., GATTER, K.C., STEIN, H. & 4 others. (1984). Human lymphoid cells express epithelial membrane antigen. *Lancet*, **ii** 1124.

ELLIS, I.O., ROBINS, R.A., ELSTON, C.W., BLAMEY, R.W., FERRY, B. & BALDWIN, R.W. (1984). A monoclonal antibody, NCRC-11, raised to human breast carcinoma. 1. Production and immunohistological characterization. *Histopathology*, **8**, 501.

FOSTER, C.S., EDWARDS, P.A.W., DINSDALE, E.A.D. & NEVILLE, A.M. (1982). Monoclonal antibodies to human mammary gland. *1 Virchows Archiv. A. Pathol. Anat. Histol.*, **394**, 279.

GATTER, K.C., ABDULAZIZ, Z., BEVERLEY, P. & 10 others. (1982). Use of monoclonal antibodies for the histopathological diagnosis of human malignancy. *J. Clin. Pathol.*, **35**, 1253.

GATTER, K.C., ALCOCK, C. HERYET, A. & 4 others. (1984*a*). The differential diagnosis of routinely processed anaplastic tumours using monoclonal antibodies. *Am. J. Clin. Pathol.*, **82**, 33.

GATTER, K.C., ALCOCK, C., HERYET, A. & MASON, D.Y. (1985). The clinical importance of analysing tumours of uncertain origin by immunohistological techniques. *Lancet*, **i**, 1302.

GATTER, K.C., FALINI, B. & MASON, D.Y. (1984*b*). The use of monoclonal antibodies in histopathological diagnosis. In *Recent Advances in Histopathology, Vol. 12*. Anthony & MacSween (eds) p.35.

GHOSH, A.K., ERBER, W.N., HATTON, C. & 4 others. (1985). Detection of metastatic tumour cells in routine bone marrow smears by immuno-alkaline phosphatase labelling with monoclonal antibodies. *Br. J. Haematol.* (in press).

HEYDERMAN, E., STEELE, K. & ORMEROD, M.G. (1979). A new antigen on the epithelial membrane: Its immunoperoxidase localisation in normal and neoplastic tissue. *J. Clin. Pathol.*, **32**, 35.

HEYDERMAN, E., STRUDLEY, I., POWELL, G., RICHARDSON, T.C., CORDELL, J.L. & MASON, D.Y. (1985). A new monoclonal antibody to epithelial membrane antigen (EMA): A comparison of its immunocytochemical reactivity with polyclonal anti-EMA antibodies and with another monoclonal antibody, HMFG2. *Br. J. Cancer*. (This issue).

LAEMMLI, U.K. (1970). Cleavage of structural proteins during assembly of the head of bacteriophage T4. *Nature* **277**, 680.

MASON, D.Y., CORDELL, J.L. & PULFORD, K.A.F. (1983). Production of monoclonal antibodies for immunocytochemical use. In *Techniques in Immunocytochemistry, Vol II*, Bullock & Petrusz (eds) p. 175. Academic Press: New York.

ORMEROD, M.G., STEELE, K., WESTWOOD, J.H. & MAZZINI, M.N. (1983). Epithelial membrane antigen: Partial purification, assay and properties. *Br. J. Cancer*, **48**, 533.

REDDING, W.H., COOMBES, R.C., MONAGHAN, P. & 8 others. (1983). Detection of micrometastases in patients with primary breast cancer. *Lancet*, **ii**, 1271.

SLOANE, J.P., ORMEROD, M.G., IMRIE, S.F. & COOMBES, R.C. (1980). The use of antisera to epithelial membrane antigen in detecting micrometastases in histological sections. *Br. J. Cancer*, **42**, 392.

SLOANE, J.P. & ORMEROD, M.G. (1981). Distribution of epithelial membrane antigen in normal and neoplastic tissues and its value in diagnostic tumour pathology. *Cancer*, **47**, 1786.

SLOANE, J.P., ORMEROD, M.G., CARTER, R.L., GUSTERSON, B.A. & FOSTER, C.S. (1982). An immunocytochemical study of the distribution of epithelial membrane antigen in normal and disordered squamous epithelium. *Diag. Histopathol.*, **5**, 11.

SLOANE, J.P., HUGHES, F. & ORMEROD, M.G. (1983). An assessment of the value of epithelial membrane antigen and other epithelial markers in solving diagnostic problems in tumour histopathology. *Histochem. J.*, **15**, 645.

TAYLOR-PAPADIMITRIOU, J., BURCHELL, J. & CHANG, S.E. (1983). Use of antibodies to membrane antigens in the study of differentiation and malignancy in the human breast. In *Monoclonal Antibodies and Cancer*, Boss *et al.* (eds) p. 227, Academic Press: London and New York.

TAYLOR-PAPADIMITRIOU, J., BURCHELL, J., MOSS, F. & BEVERLEY, P. (1985). Monoclonal antibodies to epithelial membrane antigen and human milk fat globule define epitopes expressed on other molecules. *Lancet*, **i**, 458.

TO, A., COLEMAN, D.V., DEARNALEY, D.P., ORMEROD, M.G., STEELE, K. & NEVILLE, A.M. (1981). Use of antisera to epithelial membrane antigen for the cytodiagnosis of malignancy in serous effusions. *J. Clin. Pathol.* **34**, 1326.

TO, A., DEARNALEY, D.P., ORMEROD, M.G., CANTI, G. COLEMAN, D.V. (1982). Epithelial membrane antigen: Its use in the cytodiagnosis of malignancy in serous effusions. *Am. J. Clin. Pathol.*, **78**, 214.

TOWBIN, H., STAEHLIN, T. & GORDON, J. (1979). Electrophoretic transfer of proteins from polyacrylamide gels to nitrocellulose sheets. *Proc. Natl Acad. Sci.*, **76**, 4350.

WELLS, CA.A., HERYET, A., GATTER, K.C & MASON, D.Y. (1984). The immunohistological detection of axillary lymph node micrometastatases in breast cancer. *Br. J. Cancer*, **50**, 193.